# Editorial: The dynamic plant cell wall: sensing, remodelling, and integrity

**DOI:** 10.3389/fpls.2025.1672522

**Published:** 2025-08-18

**Authors:** Laura Bacete

**Affiliations:** ^1^ Department of Plant Physiology, Umeå Plant Science Centre, Umeå University, Umeå, Sweden; ^2^ Institute for Biology, Faculty of Natural Sciences, Norwegian University of Science and Technology, Trondheim, Norway

**Keywords:** mechanosensing, cell wall integrity signalling, expansin, pectin methylesterase (PME), transcriptional regulation, Xylan (hemicellulose), drought, resilience

Plant cell walls are complex composite structures that must balance mechanical strength with plasticity (Delmer et al., 2024). They determine cell shape, provide protection against pathogens and environmental stresses, and serve as key interfaces for communication and perception. Once viewed as rigid exoskeletons, walls are now recognised as dynamic entities, whose composition and mechanics are modulated in response to developmental cues and environmental changes (Bacete et al., 2018; Gigli-Bisceglia et al., 2022; Bacete and Mélida, 2023). Advances in imaging, biophysics, and molecular biology have enabled researchers to dissect the structural and regulatory complexity of wall components—cellulose, hemicelluloses, pectins, and associated proteins—and to explore their roles in cell growth, organ development, and stress tolerance (Cosgrove, 2022).

**Figure 1:**
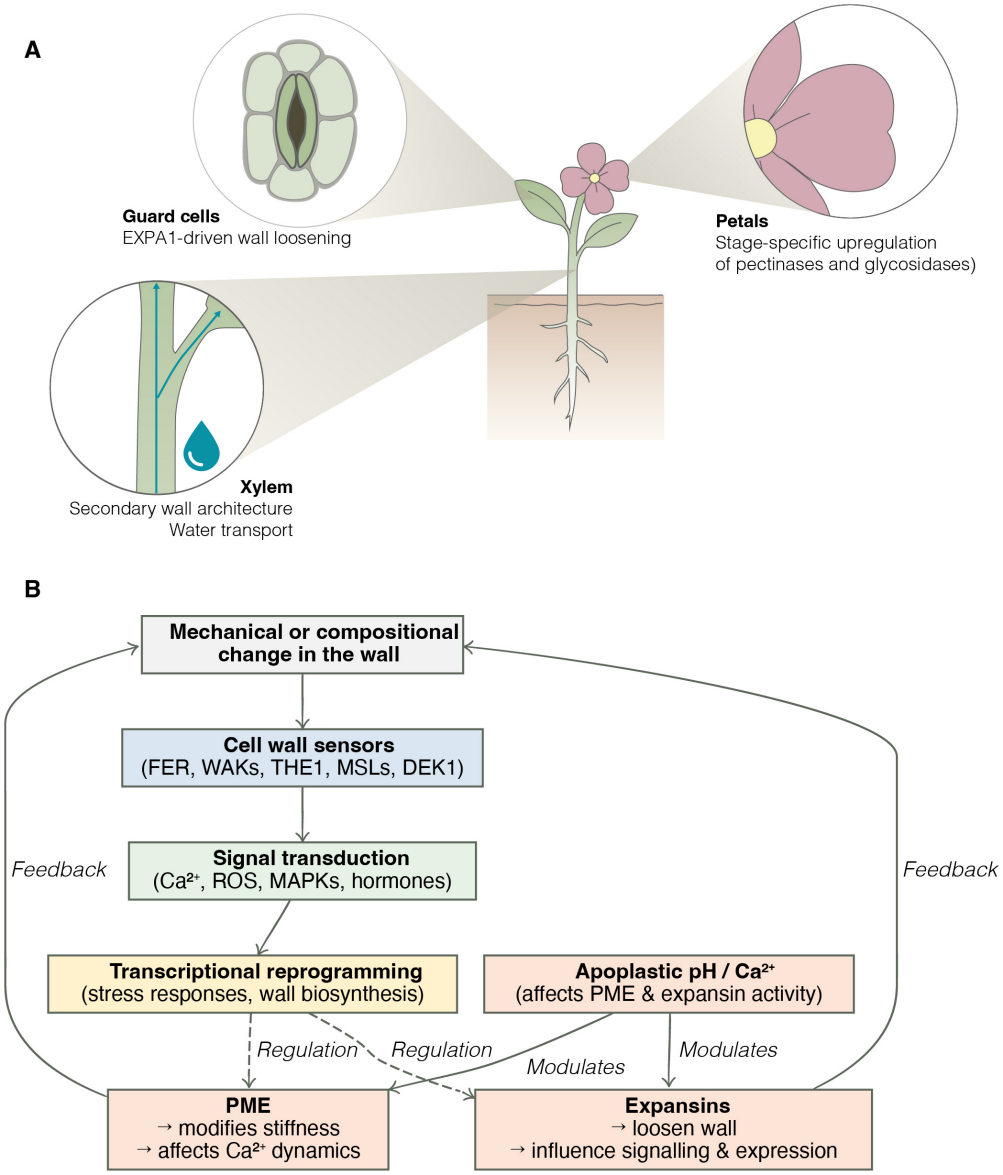
The plant cell wall as a regulatory interface. **(A)** Examples of cell wall remodelling events in distinct tissues from the five studies in this collection. EXPA1 overexpression in guard cells affects stomatal function and stress response. In xylem tissues, altered secondary wall architecture changes water transport and drought tolerance. In petals, expansion is associated with upregulated activity of wall-modifying enzymes. **(B)** Simplified model of wall sensing and feedback. Mechanical or compositional changes in the wall are perceived by wall-associated sensors such as FERONIA (FER), WALL ASSOCIATED KINASES (WAKs), THESEUS1 (THE), Mechanosensitive channel of Small conductance-Like proteins (MSLs), and DEFECTIVE KERNEL1 (DEK1). Downstream signalling modulates both wall-remodelling enzymes (e.g. PME, expansins) and transcriptomic responses. Feedback loops from apoplastic pH and calcium status further modulate PME and expansin activity, creating a context-dependent regulatory network.

The studies presented in this Research Topic illustrate a shared conceptual advance in plant cell wall biology: the wall operates not simply as a structural framework but as a responsive system, integrating mechanical, biochemical, and developmental inputs (**Figure 1**). While each paper addresses a distinct cell wall component, their findings collectively reveal how cell wall properties are monitored, modulated, and coupled to physiological outcomes.

A central theme is the conditional nature of wall remodelling. Gallemí et al. show that pectin methylesterase (PME) activity can either soften or stiffen the cell wall of *Arabidopsis thaliana* hypocotyls depending on calcium availability, emphasising the importance of local ionic context in determining mechanical output. This duality aligns with previous observations that pectin de-esterification supports both growth and reinforcement, depending on downstream modifications (Peaucelle et al., 2011; Hocq et al., 2017). In the study by Gallemí et al., PME overexpression altered elongation in the hypocotyl epidermis and demonstrated that apoplastic acidification is necessary and sufficient for cell wall softening.

Similarly, Balkova et al. demonstrate that EXPA1, an α-expansin enriched in *A. thaliana* guard cells, promotes wall loosening while also inducing transcriptional changes associated with improved drought performance. These include altered expression of other wall-modifying enzymes and improvements in photosynthetic performance, such as increased Fv/Fm (a measure of the maximum efficiency of photosystem II photochemistry) and reduced non-photochemical quenching (NPQ, which reflects the dissipation of excess energy as heat) under drought stress. These results suggest that the effects of wall-loosening proteins extend beyond mechanical changes, potentially reprogramming stress responses through cell-specific pathways.

Whereas PME and expansins act on polymer interactions within the wall, Novaković et al. focus on regulation at the level of polymer synthesis. Their study implicates the mechanosensitive protease DEFECTIVE KERNEL1 (DEK1; Amanda et al., 2016) in controlling cellulose biosynthesis and microfibril organisation in the primary walls of *Arabidopsis thaliana* cotyledon epidermal cells. DEK1 affects cellulose synthase complex activity and wall stiffness in cotyledon epidermis, possibly via indirect interaction with biosynthetic regulators. The mechanosensory role of DEK1 supports an emerging model in which wall mechanical status is not only a consequence of biosynthetic activity but also a regulator of it.

The study by Barbut et al. extends these ideas to secondary walls, focusing on xylan-deficient mutants in *A. thaliana* and *Populus tremula* L. x *tremuloides* Michx. (hybrid aspen). Altering xylan content affected drought tolerance and xylem function in both species, in some cases through compensatory lignin deposition. These findings are consistent with previous reports showing that mutations in lignin or hemicellulose biosynthesis affect vascular integrity and stress adaptation (Brown et al., 2005; Derba-Maceluch et al., 2020; Chaudhari et al., 2024). What distinguishes this study is the implication that modifying structural polysaccharides in mature tissues can shift plant hydraulic behaviour and resilience, linking wall architecture to water transport efficiency. However, the data also indicate variability across genotypes and species, suggesting that wall flexibility may enhance resilience in some tissues or conditions, potentially mediated through cell wall integrity (CWI) signalling, but may also affect growth or conductance differently.

Önder et al., examining petal development in *Rosa damascena*, provide a contrasting developmental perspective. Their findings on stage-specific shifts in wall composition and enzymatic activity underscore that similar mechanisms underlie expansion in both vegetative and reproductive tissues. Enzyme activity peaked before flower opening and dropped thereafter, indicating tight temporal control. The marked upregulation of pectinases and glycosidases during petal growth mirrors the patterns seen in vegetative organs undergoing elongation, suggesting that coordinated wall loosening is a general requirement for rapid tissue expansion (Zhang et al., 2019; Lapointe et al., 2025).

Across the five studies, a recurrent feature is the spatial and temporal specificity of wall regulation. PME and expansin activity vary by tissue type and developmental stage, while the effects of wall composition on physiology differ between primary and secondary walls. This specificity implies the existence of tightly controlled regulatory systems capable of sensing wall status and adjusting activity accordingly. The CWI signalling network, involving different sensors (**Figure 1B**), has been proposed to mediate such responses, although how it integrates with hormonal cues and mechanical feedback remains incompletely understood (Bacete and Hamann, 2020).

A key open question is how local mechanical or compositional changes in the wall produce coordinated, system-level responses. For example, the connection between EXPA1 activity in guard cells and global drought resilience is not straightforward, suggesting signalling between tissues or feedback through hydraulic or hormonal pathways (Balkova et al.). Similarly, whether xylan deficiency triggers systemic transcriptomic reprogramming or operates via local mechanical alterations remains unclear (Barbut et al.). Prior studies have documented transcriptional shifts in response to wall perturbation (Hematy et al., 2007; Denness et al., 2011), but the mechanisms by which such responses are coordinated across tissues require further investigation. Advances in spatial transcriptomics and cell-type-resolved imaging may offer tools to address this challenge.

Another area requiring clarification is how plants distinguish between mechanical signals associated with normal development and those indicating damage or stress. The same wall-modifying enzymes can participate in both processes, and the contextual determinants of their activity (whether driven by external cues, developmental programming, or internal mechanical states) remain poorly defined. This ambiguity is reflected in the dual roles of PME and expansins (Peaucelle et al., 2011; Cosgrove, 2015), and in the contrasting outcomes of secondary wall alteration, which may promote flexibility or lead to structural failure depending on context (Sivan et al., 2025).

These studies point towards the need for integrative models that link wall structure to mechanical performance and signal transduction. These relationships are summarised in the feedback model shown in **Figure 1B**, which integrates sensing, transcriptional regulation, and local feedback effects. While the current body of work provides molecular detail, it remains difficult to predict how specific wall alterations will affect growth or stress tolerance. Future progress will depend on combining biochemical and biophysical analyses with whole-plant physiological models (Alonso Baez and Bacete, 2023). Systems that allow spatial and temporal control of wall remodelling, such as inducible expression or tissue-specific promoters, could help establish causal relationships between local wall properties and emergent phenotypes. At present, few models incorporate mechanical feedback, limiting predictive capacity.

Taken together, the findings in this Research Topic emphasize that the plant cell wall is not simply a product of growth but a regulator of it. Its composition and mechanical status influence development, signal perception, and environmental response. By dissecting the interplay between structure and function, these studies advance our understanding of how walls shape plant adaptation.
